# Exosomal ITGA3 interferes with non-cancerous prostate cell functions and is increased in urine exosomes of metastatic prostate cancer patients

**DOI:** 10.3402/jev.v2i0.22097

**Published:** 2013-12-23

**Authors:** Irene V. Bijnsdorp, Albert A. Geldof, Mehrdad Lavaei, Sander R. Piersma, R. Jeroen A. van Moorselaar, Connie R. Jimenez

**Affiliations:** 1 Department of Urology, VU University Medical Center, Amsterdam, The Netherlands; 2 OncoProteomics Laboratory, Department of Medical Oncology, VU University Medical Center, Amsterdam, The Netherlands

**Keywords:** prostate cancer, integrin, biomarker, proteomics, non-cancerous cells

## Abstract

**Background:**

Cancer cells are able to change the protein expression and behavior of non-cancerous surrounding cells. Exosomes, secreted by prostate cancer (PCa) cells, may have a functional role in cancer metastasis and present a promising source for protein biomarkers. The aim of the present study was to identify which proteins in exosomes can influence non-cancerous cells, and to determine whether we can use urine exosomal proteins to identify high-risk PCa patients.

**Method:**

Exosomes were isolated by ultracentrifugation. Migration and invasion were studied by the transwell (invasion) assay. Proteomics was performed by LC-MS/MS and identified proteins were validated by Western blotting. Cellular uptake of fluorescent labeled PKH67-exosomes was measured by FACS.

**Results:**

Based on comparative protein profiling by mass spectrometry-based proteomics of LNCaP- and PC3-exosomes, we selected ITGA3 and ITGB1, involved in migration/invasion, for further analyses. Inhibition of exosomal ITGA3 reduced the migration and invasion of non-cancerous prostate epithelial cells (prEC) almost completely. Cellular uptake of exosomes by prEC was higher with PC3-exosomes compared to LNCaP exosomes. Finally, ITGA3 and ITGB1 were more abundant in urine exosomes of metastatic patients (p<0.05), compared to benign prostate hyperplasia or PCa.

**Conclusion:**

These data indicate exosomal ITGA3 and ITGB1 may play a role in manipulating non-cancerous surrounding cells and that measurement of ITGA3 and ITGB1 in urine exosomes has the potential to identify patients with metastatic PCa in a non-invasive manner.

Prostate cancer (PCa) is the most common diagnosed cancer and the second cancer-related cause of death among Western men. Fatal outcome from PCa is generally preceded by invasive and metastatic growth (1). However, the majority of the PCas will never progress and become metastatic (2, 3). Current prognostic markers consist of serum levels of prostate-specific antigen (PSA), Gleason score and pathological stage (4). However, with these tests, it is not possible to accurately distinguish indolent from more aggressive cancer. The implementation of novel state-of-the-art technologies such as proteomics has led to the discovery of many more potential protein biomarkers not only in tissues but also in serum and urine. However, serum proteomics is challenging because high-abundant proteins may mask low-abundant proteins, and tissue-based proteomics may be hampered due to the fact that it is hard to predict which tissue proteins may provide reliable biofluid-based markers. Small microvesicles (exosomes), secreted by (cancer) cells may be considered to be a pseudo tissue fraction in biofluids and therefore may provide a promising alternative avenue for discovery of novel non-invasive candidate protein biomarkers for diagnosis and disease stratification. Also, when urine is used for diagnosis, exosomes will overcome the limitation of the large variety in volume and dilution of the biomarker (5).

Exosomes are small membrane vesicles, about 100 nm in size. They are derived from the luminal membranes of multivesicular bodies and are actively released by fusion of the multivesicular bodies with the cell membrane. Due to the fact that exosomes are secreted by prostate cells, they can be found in serum and urine, which makes these exosomes very attractive for the discovery of novel biomarkers (6). Exosomes are thought to play a role in local and systemic communication through horizontal transfer of molecules such as RNA and proteins. A recent report shows strong evidence for this systemic communication (7), providing additional support for the functionality of exosomes in cancer development and progression. However, the exact mechanism of the underlying exosomal cross-talk between cancer and non-cancerous cells remains to be elucidated. It is unknown what effect the exosomes derived from cancer cells have on the (adjacent) non-cancerous cells.

Previously, we reported that a change in connexin-26 (a protein involved in intercellular communication) expression in tumour surrounding non-cancerous tissues was related to metastatic tumour (8). In addition, we found that conditioned medium (secretome) from metastatic PCa cells increased the migration and invasion of non-cancerous prostate epithelial cells (prEC). To understand more about these influences of cancer cells on their non-cancerous surrounding cells, we hypothesized that exosomes secreted by PCa cells contain proteins that influence the behaviour of surrounding non-cancerous epithelial cells. Therefore, in this study, with proteomic analysis of cancer exosomal proteins, we identified potential proteins that were involved in migration and invasion (Fig. 1A). We show clear evidence that cancer-derived exosomes have a function in changing non-cancerous cell behaviour. Due to this changed healthy cell behaviour, cancer cells with more aggressive growth may be able to create a microenvironment that stimulates progression and even distant metastasis. More importantly, as a proof of concept, we have identified the proteins ITGA3 and ITGB1 to be significantly more abundant in urine of mPCa compared to benign prostate hyperplasia (BPH) and PCa patients. These data indicate that the approach to use urine exosomes from PCa patients has great potential for risk stratification of patients with high serum levels of PSA.

**Fig. 1:**
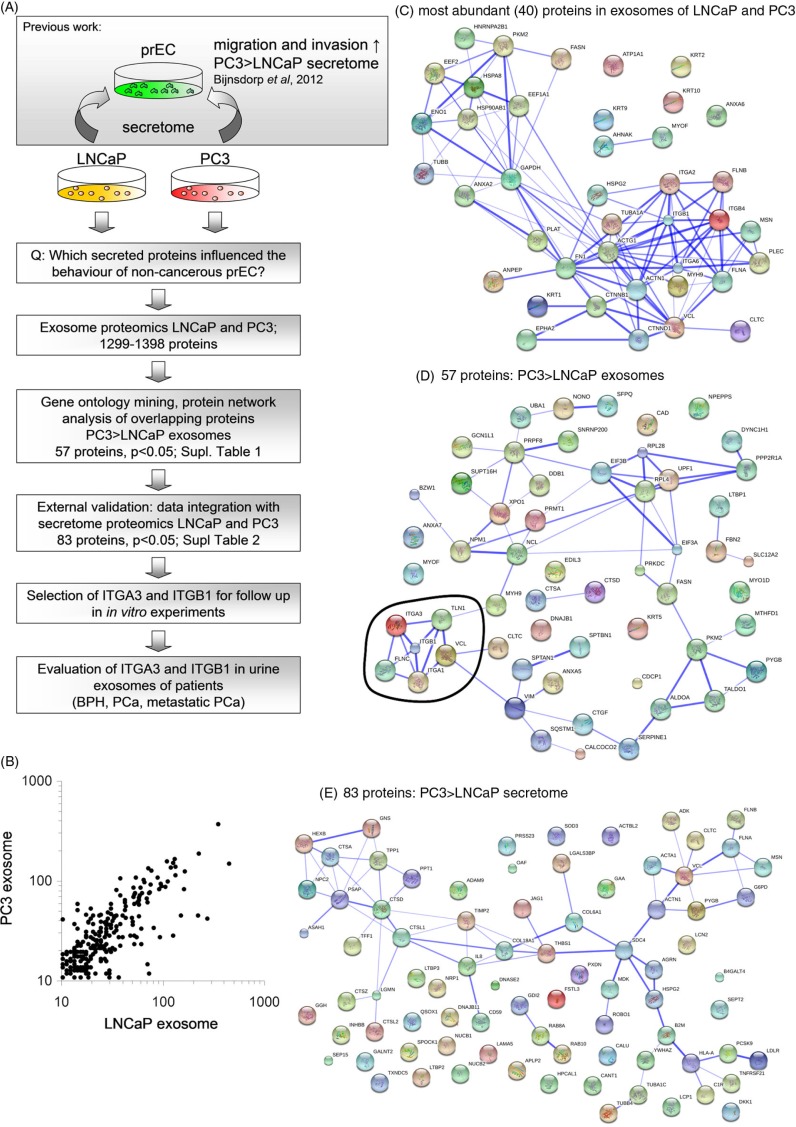
**(A)** Study workflow diagram. This diagram gives an overview of the experimental steps performed in this study. **(B)** Scatter plot of comparison of expression levels of identified proteins in LNCaP and PC3 exosomes. **(C)** String analysis of proteins present (>50 times detected) in both LNCaP and PC3 exosomes. **(D)** String analysis of proteins that were abundant at significantly (p<0.05) higher levels PC3 exosomes compared to LNCaP exosomes. All proteins were abundantly expressed in the exosomes of both cell lines. In this analysis, a cluster of proteins (encircled) was involved in migration and invasion (David functional analysis). **(E)** String analysis of proteins that were abundant at significantly (p<0.05) higher levels PC3 secretome compared to LNCaP secrectome. Secretome data were obtained from Sardana et al., 2008 (15).

## Materials and methods

### Cell lines and antibodies

The human PCa cell lines LNCaP and PC3 (obtained from the American Type Culture Collection, Rockville, MD) were cultured *in vitro* in RPMI 1640 supplemented with 10% FCS and 50 U/ml penicillin and 50 µg/ml streptomycin. For a model of non-cancerous prEC, we used prEC (CC-2555) obtained from Lonza (Verviers, Belgium). These cells show characteristics of non-cancerous prostate cells but lack expression of PSA and androgen receptor. prEC cells were cultured according the manufacturer's instructions. In brief, prEC were cultured in PrEBM basal medium (Lonza, CC-3165), with supplements and growth factors (prEGM SingleQuot Kit, Lonza, CC-4177). The medium was refreshed every day, to maintain cellular growth. Cells were used between passages 3 and 5. All cells were maintained in a 5% CO_2_-humidified atmosphere at 37°C. Anti-ITGA3 antibody was obtained from Abcam (Cambridge, UK), and antibodies against ITGB1 (#4706) and Alix (PDCD6IP; #2171) were obtained from Cell Signaling Technology (Danvers, MA).

### Exosome isolation from cell lines

LNCaP (1.5×10^6^ cells) and PC3 (1×10^6^ cells) were seeded in T75 culture flasks. After 24 hours, medium was replaced by serum-free medium. After another 48-hour incubation, the medium was collected and centrifuged for 5 minutes at 1,500 rpm to remove any cells, followed by 20,000×g for 20 minutes at 4°C to remove any debris. Exosomes were isolated by ultracentrifugation at 100,000×g for 90 minutes at 4°C (2×).

### Proliferation assay

prEC cells were seeded (5,000 cells/well) in 96-well plates (Greiner BioOne, Frickenhausen Germany). Twenty-four hours after seeding, the medium was replaced by fresh culture medium, with and without diluted fractions of the concentrated conditioned medium derived from LNCaP or PC3 cells. At t=1 day, t=2 days, the medium was refreshed, containing the conditioned medium. At t=3 days, the cells were fixed by trichloroacetic acid at 4°C for 1 hour and stained by sulphorhodamine B (SRB) as described previously (9). The SRB dye was diluted in 10 mM Tris solution and the optical density (OD) was measured at 540 nm after which the OD of the controls was set to 100%.

### Migration and invasion assay

The migration and invasion assays were carried out as described previously (8, 9). In brief, transwell chambers with a fluorescence-blocking 8-µm pore filter insert (#35- 1152; HTS Fluoroblok Insert, Falcon, Becton Dickinson Labware, Bedford, MA) were used. For measuring the invasion, the insert was coated overnight at room temperature (RT) with 100 µl matrigel (50 ng/ml in PBS; Sigma). Cells (200,000/insert) were seeded in serum-free medium containing conditioned medium or exosomes from LNCaP and PC. In the lower chamber, normal culture medium was added together with the matching conditioned media or exosomes. Cells were allowed to invade for 8 hours. Calcein-AM (5 µM) was added 30 minutes before analysis to the lower chamber and fluorescently labelled cells were counted.

### Proteomics sample preparation

For proteomics, exosomes were isolated as described above and lysed in reducing sodium dodecyl sulphate (SDS) sample buffer (62.5 mM Tris-HCl, 2% w/v, SDS, 10% v/v glycerol, and 0.0025% bromophenol blue, 100 mM DTT, pH 6.8). Proteins were heated at 100°C for 10 minutes and separated by SDS-polyacrylamide gel electrophoresis. Each sample (containing about 50 µg protein) was loaded on a pre-cast NuPAGE 4–12% w/v Bis-Tris 1.5-mm minigel (Invitrogen, Carlsbad, CA). Replicate analysis has shown good reproducibility of the entire workflow from exosome isolation to protein identification and quantification (10, 11). Gels were fixed (50% ethanol and 3% phosphoric acid) and stained (Coomassie Blue G-250, BioRad, Hercules, CA). Gel lanes of each sample were divided into 10 bands, and each band was processed for in-gel digestion as described before (12).

### LC-MS/MS

Peptides were separated using an Ultimate 3,000 nanoLC system (Dionex LCPackings, Amsterdam, The Netherlands) as described before (10). Chromatographic separation of peptides was performed by a 68-minute gradient at 300 nl/minute. After injection, peptides were trapped at 6 µl/min on a 15 mm×100 µm ID trap column packed with 5 µm 100 Å ReproSil Pur C18 aqua (Dr Maisch GMBH, Ammerbuch-Entringen, Germany) at 2% buffer B (buffer A: 0.05% formic acid in MQ; buffer B: 80% ACN+0.05% formic acid in MQ) and separated at 300 nl/min in a 10–40% buffer B gradient for 60 minutes (90 minutes inject-to-inject). Eluting peptides were ionized at 1.7 kV in a Nanomate Triversa Chip-based nanospray source using a Triversa LC coupler (Advion, Ithaca, NJ). Intact peptide mass spectra and fragmentation spectra were acquired on an LTQ-FT hybrid mass spectrometer (Thermo Fisher, Bremen, Germany). Intact masses were measured at resolution 50,000 in the ICR cell using a target value of 1×10^6^ charges. In parallel, following an FT pre-scan, the top 5 peptide signals (charge states 2+ and higher) were submitted to MS/MS in the linear ion trap (3 amu isolation width, 30-ms activation, 35% normalized activation energy, Q value of 0.25 and a threshold of 5,000 counts). Dynamic exclusion was applied with a repeat count of 1 and an exclusion time of 30 seconds.

### Proteomic data analysis

Protein identification was performed as described previously, MS/MS spectra were searched against the human IPI database 3.59 (80,128 entries) using Sequest (version 27, rev 12), which is part of the BioWorks data analysis package (Thermo Fisher, San Jose, CA). MS/MS spectra were searched with a maximally allowed deviation of 10 ppm for the precursor mass and 1 amu for fragment masses. Scaffold was used to organize the gel-band data and to validate peptide identifications using the Peptide Prophet algorithm. Only identifications with a probability >95% were retained. Subsequently, the Protein Prophet algorithm was applied, and protein identifications with a probability of >99% with 2 peptides or more were retained in at least 1 sample. Using these stringent peptide and protein identification criteria, the FDR is estimated at ±0.5%, as calculated previously (13). Normalization was performed as described previously (10). The spectral counts of each protein were divided by the total spectral counts of all proteins within a sample. This number was multiplied with a constant equal to the average of total spectral counts of all samples to obtain a normalized spectral count value in the same range as the non-normalized spectral counts. The beta-binomial test was applied to find proteins that show significant differences in spectral count numbers between the tumour group and the reference group. Proteins with a p-value <0.05 were designated as being significant. For analysis of pathways involved, we used String database and David functional analysis.

### Western blotting

Western blotting was performed as described previously (14). In brief, cells were stimulated with conditioned medium for 6 hours, washed with PBS and scraped in lysis buffer (Cell Signalling Technology Inc., suppl. with 0.04% protease inhibitor cocktail). For evaluating protein expression in the exosomes, exosomes were isolated by ultracentrifugation and directly lysed in lysis buffer. After centrifugation at 11,000 g at 4°C, protein amounts in the supernatants were determined by the Bio-Rad assay (Bio-Rad Laboratories, Veenendaal, The Netherlands). A total of 40 µg of protein was separated on a 10% SDS-PAGE and electroblotted onto polyvinylidene difluoride (PVDF) membranes (Millipore Immobilon^TM^ – FL PVDF, 0.45 µm). Subsequently, the membranes were blocked and incubated overnight at 4°C with the primary antibodies (dilution 1:1,000–10,000) and subsequently with the corresponding secondary antibody against mouse or rabbit (goat-α-mouse-IRDye (680; #926-32220) or goat-α-rabbit-IRDye (800CW;926-32211), Westburg, Leusden, The Netherlands). The bands were scanned using an Odyssey Infrared Imager (Westburg) with high quality and analyzed using Odyssey software programme, of which the intensity was corrected for PDCD6IP (exosomes) or β-actin (cells) expression levels.

### FACS analysis of exosomal uptake

Exosomes were labelled for 3 minutes with PKH67 dye in Diluent C (# PKH67GL, Sigma-Aldrich chemicals, Zwijndrecht, The Netherlands) in dark, according to the manufacturer's protocol. As negative controls, RPMI without cellular exosomes was used. Subsequently, to remove access dye, exosomes or PBS was centrifuged for 1 hour at 100,000×g at 4°C. The exosomal pellet was then diluted in 200 µl medium. Subsequently, prEC cells were exposed to the labelled exosomes, and subsequently analyzed by FACS for PKH67 uptake (FL-1).

### Urine sample collection and exosome isolation

Urine from patients with BPH (n=5), PCa (n=5) and metastatic PCa (n=3) were selected for evaluation of protein expression in the exosomes. All samples were collected and stored in equal conditions at −80°C until exosome isolation. Five milliliters of urine was subjected to serial centrifugation, removing cells (2,000×g, 15 minutes), and removing non-cellular debris (10,000×g, 30 minutes). The supernatant was then centrifuged at 100,000×g for 1.5 hours at 4°C, washed in PBS and subsequently centrifuged again at 100,000×g for 1.5 hours. Exosome pellets were resuspended in 100 µl lysis buffer (Cell Signaling Technology, 0.04% protease inhibitor cocktail) and subjected for Western blotting as described above.

### Statistical analyses

Potential differences between samples were evaluated using the two-tailed Student's t-test for paired or unpaired data. Changes were considered significantly different when p<0.05.

## Results

### Proteomic identification of ITGA3 and ITGB1 in exosomes as migration/invasion-related proteins

Previously, we showed that PC3 (hormone-independently growing, more aggressive) and LNCaP (hormone dependently, less aggressive) conditioned medium (containing exosomes and soluble proteins) could increase the proliferation, migration and invasion of non-cancerous epithelial cells (8). To select potential proteins that are present in the secretome of PC3 and LNCaP that are able to change the behaviour of these non-cancerous cells, we evaluated which proteins were present in the medium of these cell lines by analyzing proteomics data previously published by Sardana et al. (15). In addition, in view of the importance of exosomes in intracellular communication, we identified and quantified proteins present in exosomes that are secreted by LNCaP and PC3 cells, using proteomics (Fig. 1A). LC-MS/MS identified in total 1,299 proteins in LNCaP exosomes and 1,398 proteins in PC3 exosomes (Supplementary file). The profile and expression levels of the exosomal proteins were highly comparable between LNCaP and PC3 (Fig. 1B). Many (>45%) of these 40 most abundant exosomal proteins were involved in adhesion, migration and cytoskeleton organization (p<0.005) (Fig. 1C).

As PC3 cells were previously able to change the behaviour of the non-cancerous cells to a higher extent (8), we also selected proteins based on a higher abundance in PC3 exosomes compared to LNCaP exosomes as determined by their spectral counts. A total of 101 proteins were significantly higher (p<0.05) expressed in PC3 exosomes, compared to those in LNCaP exosomes. Of the latter group, 57 proteins were detected in both LNCaP and PC3 exosomes (Supplementary file). Since the secretome of both PC3 and LNCaP that could potentially be involved in the changed behaviour of the non-cancerous cells, the 57 overlapping exosomal proteins were subjected to David functional analysis and String database. The top KEGG pathways were involved in focal adhesion (p<0.005) and regulation of the cytoskeleton (p<0.05). String database of predicted functional associations between proteins showed one clear cluster of protein interactions, which involved cell adhesion and motility (Fig. 1D). From these analyses based on String and David, 6 proteins were identified to play a role in migration/invasion (Fig. 1D and Table I). From these proteins, we selected ITGA3, ITGB1, TLN1 and VCL for further evaluation.

**Table I:** Protein composition of exosomes of LNCaP and PC3 cells involved in focal adhesion and cytoskeletal regulation

Gene symbol	Gene name	Expression LNCaP exosomes	Expression PC3 exosomes	Ratio	p	LNCaP* secretome	PC3* secretome	Involved in process
ITGA3	Integrin α3	35.1	80.5	2.3	0.0111	0	19.9	Cell surface adhesion molecules that undergoes posttranslational cleavage and can interact with ITGB1
ITGB1	Integrin β1	66.0	96.1	1.5	0.0436	0	79.6	Cell adhesion and migration; cell growth and survival; activation of intracellular signaling; tumor metastasis and angiogenesis
TLN1	Talin 1	23.7	44.6	1.9	0.0366	7.1	5.2	Cell–cell contacts; codistributes and interacts with integrins and other focal adhesion proteins
VCL	Vinculin	65.0	100.9	1.6	0.0293	16.6	33.7	Focal adhesion regulation; cell adhesion and motility
ITGA1	Integrin α1	5.2	19.4	3.8	0.0255	n.d.	n.d.	Cell surface adhesion molecules, heterodimerizes with the beta 1 subunit to form a cell-surface receptor for collagen and laminin
FLNC	Filamin C	19.6	52.4	2.7	0.0134	0	15.6	Involved in reorganizing the actin cytoskeleton in response to signaling events

n.d.=not detected.

* LNCaP and PC3 secretome spectral counts were obtained from Sardana et al., 2008 (15).

In the secretome from Sardana et al. 1,810 proteins were identified by nanoLC-MS/MS (15). When a selection on the downloaded data was made on higher abundance in PC3 secretome compared to LNCaP secretome using the beta-binomial test, 353 proteins were found at significantly higher levels, including 83 proteins that were overlapping between the secretome of both cell lines (Supplementary file). These 83 proteins were subjected for functional analysis by David functional analysis and String database. KEGG pathways were involved in lysosome (p<0.00001), ECM–receptor interaction (p<0.05) and focal adhesion (p<0.05). String database showed a clear cluster of protein interactions, which involved lysosome functions (Fig. 1E). The proteins that we have found in the exosomes, ITGA3, ITGB1, VCL and TLN1 were also found in the dataset of the secretome of Sardana et al. (15), and were present at significant higher levels in PC3 medium, and were not detected or present at lower levels in LNCaP medium, which is in agreement with our exosome proteomics data (Table I).

### Cellular and exosomal expression of ITGB1 and ITGA3

**Fig. 2:**
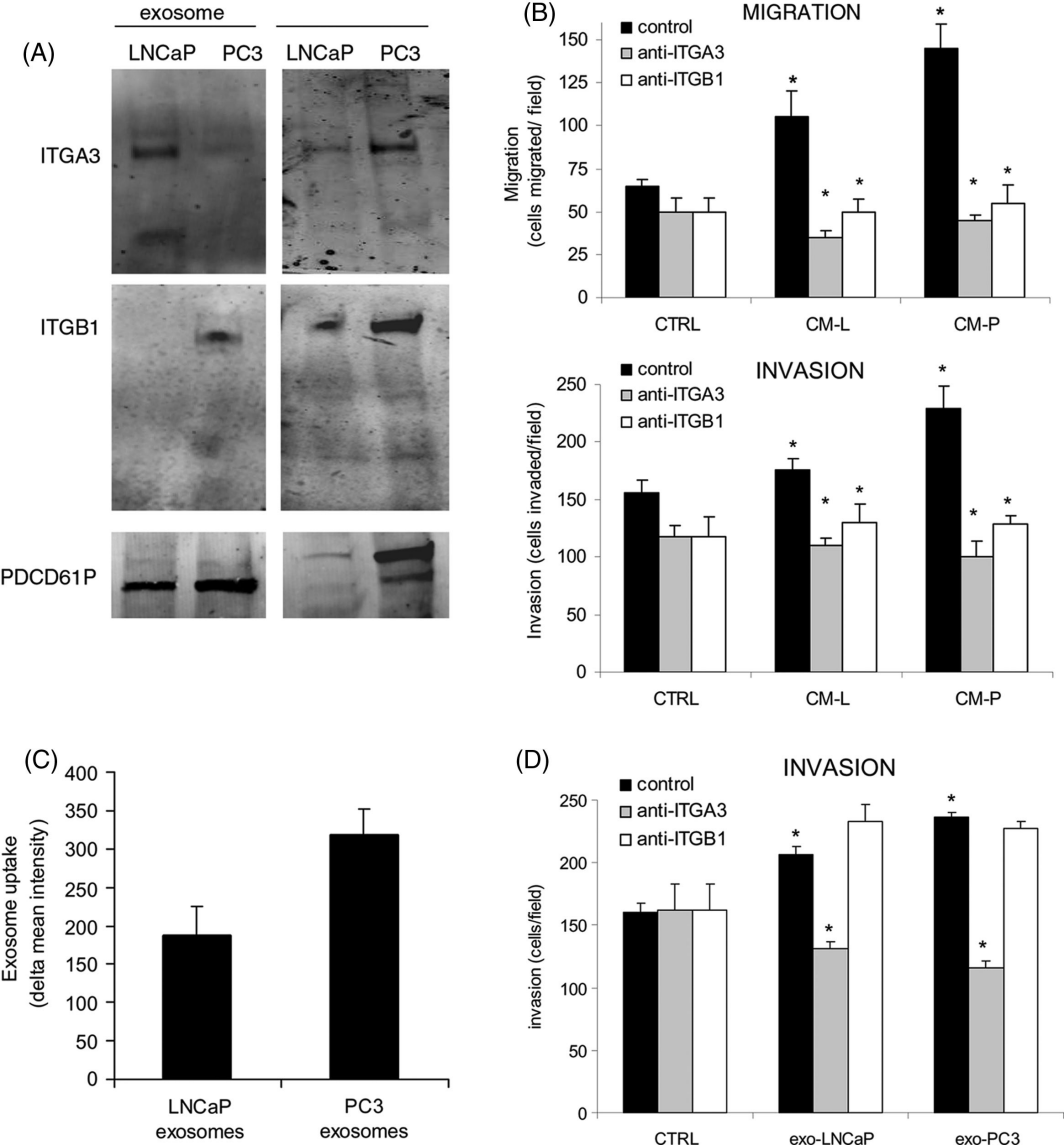
**(A)** Western blot showing the expression of ITGB1, ITGA3 and PDCD6IP in exosomes and whole cell lysates of LNCaP and PC3 cells. **(B)** Effect of blocking antibodies of ITGA3 and ITGB1, which were added combined with LNCaP-medium (CM-L) or PC3-medium (CM-P) on the migration and invasion of prEC. As negative controls, isotype control was added. Statisticial differences between controls and between antibody and CM-L or CM-P are indicated in the graph (*p<0.05) **(C)** Exosomal uptake of LNCaP and PC3 exosomes in prEC cells as measured by FACS analysis. Values represent means of 3 independent experiments. **(D)** Effect of exosomes from LNCaP and CP3 cells on prEC invasion, with/without blocking antibodies against ITGA3 and ITGB1. As antibody-controls, isotype control was added. Statisticial differences are indicated in the graph (*p<0.05).

To confirm the presence of the selected proteins in the cells and exosomes, Western blotting was performed. ITGB1 was highly expressed by both LNCaP and PC3 cells (Fig. 2A). ITGB1 was not detectable by Western blotting in LNCaP exosomes and to some extent in PC3 exosomes, which is in agreement with the proteomic analyses. ITGA3 (30kDa, a known cleaved and functional form of ITGA3) was expressed by PC3 cells, and hardly detectable in LNCaP cells. ITGA3 was present at low levels of LNCaP exosomes and, surprisingly, hardly detectable in PC3 exosomes. Because we detected the 30 kDa variant by Western blotting, we evaluated which isoforms of ITGA3 were measured by proteomics analysis, and found 2 major isoforms of ITGA3, one of ~116 kDa and one of ~30 kDa. Both kDa isoforms exhibited similar quantitative differences (1.7- vs. 1.9-fold difference) between LNCaP and PC3 exosomes. VCL was observed at very low levels in PC3 exosomes and was not detected in exosomes from LNCaP (data not shown). TLN1 was not detectable in either cells or exosomes (data not shown). Therefore, in continuing experiments, we only evaluated ITGA3 and ITGB1. In addition, we evaluated PDCD6IP expression by Western blotting as a protein known to be present in exosomes (16). PDCD6IP was expressed at comparable levels in the exosomes of the 2 cell lines, confirming that the isolated vesicles are exosomes.

### Inhibition of the proteins reversed the effect on migration and invasion

To evaluate whether these selected proteins in exosomes have a function in the migration and invasion of the non-cancerous cells, we blocked ITGA3 and ITGB1 using blocking antibodies and subsequently evaluated the effect on proliferation, migration and invasion (Fig. 2B). Blocking both ITGA3 and ITGB1 caused a significant drop in migration and invasion of prEC cells (Fig. 2B). prEC cell proliferation was not changed by the addition of any of the blocking antibodies (data not shown). In addition, we evaluated whether these exosomes could increase the invasion of prEC themselves. Both exosomes from LNCaP and PC3 cells stimulated the invasion of prEC cells. Blocking ITGA3 reduced the levels to control levels, while ITGB1 antibody had no effect on the invasion of prEC (Fig. 2D). Taken together, even though the expression was low as found by Western blotting, ITGA3 and ITGB1 in conditioned medium and ITGA3 in exosomes were involved in the stimulation of migration and the invasion of the non-cancerous epithelial cells.

### Higher uptake of PC3 exosomes compared to LNCaP exosomes

To determine to what extent the exosomes were taken up by the cells, we performed FACS analyses of exosomal uptake into the non-cancerous cells. Interestingly, exosomes from PC3 cells were taken up by prECs at higher levels compared to LNCaP exosomes, respectively (Fig. 2C). Since the antibodies could block the migration/invasion function of prEC, we studied whether the identified proteins were involved in the uptake of the exosomes. Therefore, we inhibited these exosomal proteins using blocking antibodies, and subsequently determined exosomal uptake into non-cancerous cells. However, the tested blocking antibodies barely changed the exosomal uptake (data not shown), which indicates that these proteins are probably not involved in exosomal uptake by the cells.

### Levels of ITGA3 and ITGB1 are increased in urine exosomes from metastatic PCa patients

**Fig. 3:**
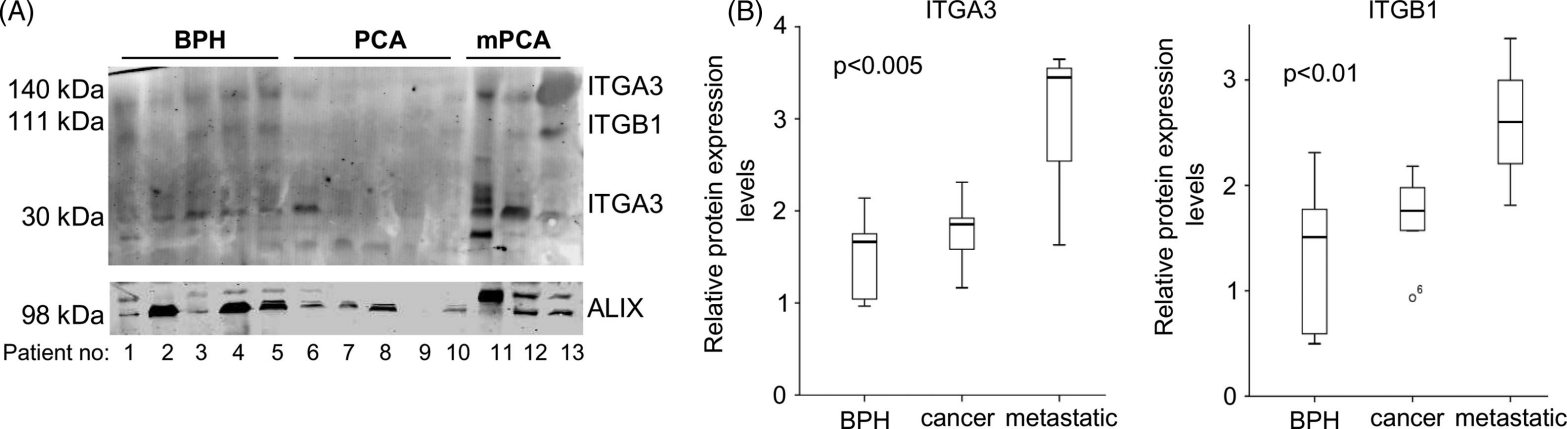
**(A)** Western blot showing the expression of ITGA3, ITGB1 and PDCD6IP in urine-exosomes of patients suffering from BPH, prostate cancer (PCA) and metastatic prostate cancer (mPCA). **(B)** Quantitative analysis showing the boxplots of the expression of ITGA3 (30kDa variant) and ITGB1, relative to PDCD6IP.

Exosomes derived from prostate (cancer) cells have been reported to be detectable in the urine (5). Therefore, we investigated whether we could measure the identified proteins in exosomes from urine of patients with BPH, PCa and metastatic PCa by Western blotting. We were unable to isolate equal amounts of protein from the urine exosomes, since we had a small volume (5 ml urine). Therefore, we loaded equal volumes, and used PDCD6IP as the loading control (Fig. 3). ITGA3 was visible at 2, 140 and 30 kDa heights, respectively. The 140-kDa ITGA3 isoform was detectable at low levels in most urine samples. However, the 30-kDa ITGA3 variant was detectable in BPH samples, hardly in PCa samples and highly detectable in 2 of the 3 metastatic PCa samples. ITGB1 was visible at very low levels in BPH, not detectable in cancer and detectable at somewhat higher levels in metastatic PCa. When corrected for PDCD6IP levels, ITGA3 (30 kDa) and ITGB1 were significantly higher expressed in metastatic PCa, compared to BPH and PCa (Fig. 3B). Although the number of patients was limited, these data indicate that using urine exosomes for low-invasive diagnosis of (metastatic) PCa holds great potential.

## Discussion

Accumulating evidence indicates that exosomes can act as mediators of tumour progression and metastasis. A recent study showed that serum-exosomes can create a prometastatic niche at sites by horizontal transfer of exosomal proteins (7). In this study, we used proteomics to identify exosomal proteins that potentially modulate the behaviour of non-cancerous prECs. The proteins ITGA3 and ITGB1 were abundant in cancer cell exosomes and in the secretome of metastatic cell lines. Importantly in an exploratory analysis by Western blot, we show that these proteins were more abundant in urine exosomes of metastatic PCa compared to PCa and BPH, which underlines the great potential for using exosomal proteins for risk stratification.

For indolent PCa, no therapy is required. However for aggressive and metastatic PCa, early treatment is mandatory. When a cancer has metastasized, therapy will be palliative due to the highly resistant phenotype of PCa. Identification of candidate markers for aggressive (metastatic) PCa has been very difficult, partially due the high heterogeneity of PCa. As exosomes are secreted by cancer cells and therefore may contain various tumour-specific proteins and RNAs, exosomes are attractive to use in non-invasive diagnostic approaches. The knowledge about the role of exosomes in cancer metastasis is increasing (17–20). The underlying molecular mechanisms of exosomes and metastasis and whether we can use these for non-invasive diagnostic tests remains to be investigated in further studies.

Our proteomic analysis identified several proteins with increased levels in metastatic exosomes that were involved in migration and invasion processes. ITGA3 and ITGB1 are integrin proteins. Several integrins have previously been identified in exosomes both from urine and derived from cancer cell lines, including integrin VLA-4, integrin α3, integrin αM, integrin β1 and integrin β2 (14, 21–27). This indicates that probably all exosomes express these integrins and that this expression increases to higher levels when metastasis develops. In addition, in prostatic secretions, various proteins were found that we also observed in PC3 and LNCaP exosomes, including MYH9, LAMA5 and CTSD (28. Integrins are important for adhesion to a multitude of ligands (29). ITGA3 and ITGB1 were described to play a role in cell adhesion, cell-matrix adhesion and integrin-mediated signalling pathway (30, 31). Of ITGA3 we found the cleaved 30-kDa variant in exosomes of both tested cell lines and in urine exosomes. This variant is the light chain variant of ITGA3, since ITGA3 is cleaved posttranslationally into a light and heavy chain, that heterodimerize with ITGB1, and can then bind to the laminin-511 (32). Integrin–α3β1 complex is also known for its functions in migration and invasion and is a potential target against breast cancer (33, 34). In contrary, a high cellular ITGA3 expression has also been correlated with decreased metastasis of PCa cells (35). In literature, there is no consensus about the role of integrin–α3β1 in metastasis (36, 37). We found a higher expression in exosomes secreted by PCa cells. In future studies, more sensitive methods to detect proteins (e.g. ELISA) might give more quantitative results as the detection by Western blotting is limited. In this study, exosomal ITGA3 and ITGB1 were involved in the changed behaviour of the non-cancerous prECs, which may indicate an active role in the metastatic process. Moreover, that these integrins were differentially expressed in urine exosomes underlines the potential to use for a more accurate risk stratification for metastasis.

Different studies already indicated that the adjacent benign (normal) prostate cells can have a predictive role for the presence of cancer (8, 38) (39). A change in protein expression profile of adjacent normal prostate cells may be attributed to uptake of cancer exosomes. Previously, it has also been described that exosomes from metastatic cancer (melanoma) play a crucial role in the formation of the metastasis by educating sites to become a prometastatic environment due to vascular leakiness (7). Our study shows that non-cancerous cells stimulated by more aggressive cell-derived exosomes with altered protein composition, changed their behaviour towards a more aggressive one. Whether this changed behaviour contributes to a higher metastatic potential of the cancer cells remains to be studied in future experiments. Our data support the observation of others that exosomes have a function in intercellular communication.

In conclusion, we have identified several proteins present in cancer-secreted exosomes that play a role in the modulation of proliferation, migration and invasion capacity of non-cancerous cells. Of these proteins, ITGA3 and ITGB1 were highly expressed in urine exosomes of metastatic PCa patients, which may be used in addition to current diagnostic tests to identify metastatic PCa patients using a non-invasive test.
